# Laparoscopic injury of the obturator nerve during fertility-sparing procedure for cervical cancer

**DOI:** 10.1186/1477-7819-10-177

**Published:** 2012-08-30

**Authors:** Enzo Ricciardi, Marina Jakimovska, Paolo Maniglio, Mauro Schimberni, Antonio Frega, Borut Kobal, Massimo Moscarini

**Affiliations:** 1 Department of Obstetrics and Gynecology, Sapienza University of Rome. Sant’Andrea Hospital, via di Grottarossa, 1035-1039, Rome, 00189, Italy; 2 Department of Obstetrics and Gynecology University Medical Center, Ljubljana, Slovenia

**Keywords:** Cervical cancer, Fertility-sparing procedure, Laparoscopy, Lymphadenectomy, Obturator nerve injury

## Abstract

**Background:**

Intraoperative injury of the obturator nerve has rarely been reported in patients with gynecological malignancies undergoing extensive radical surgeries. Irreversible damage of this nerve causes thigh paresthesia and claudication. Intraoperative repair may be done by end-to-end anastomosis or grafting when achieving tension-free anastomosis is not possible.

**Case presentation:**

A 28-year-old woman with stage IB cervical cancer underwent fertility–sparing surgery, including conization and bilateral pelvic lymphadenectomy. The left obturator nerve was damaged intraoperatively during pelvic dissection.

**Conclusion:**

Immediate laparoscopic repair was successful and there was no functional deficit in the left thigh for six months postoperatively.

## Background

Obturator neuropathy is an uncommon condition presenting as medial thigh or groin pain, weakness with leg adduction, and sensory loss in the medial thigh of the affected side. Obturator nerve injury is rare in obstetrics and gynecology. Patients with gynecologic cancer, who are undergoing radical pelvic surgery and, specifically, pelvic lymphadenectomy in the obturator fossa are, however, at increased risk of obturator nerve injury [1]. Obturator nerve injury can result from sectioning, stretching or crushing of the nerve. Options for surgical management of obturator nerve injury include transabdominal, laparoscopic and extraperitoneal approaches [2,3]. Herein we report a case of obturator nerve transection during laparoscopic pelvic lymph node dissection, conization and pelvic lymphadenectomy and its immediate laparoscopic repair.

## Case presentation

We present a case of a 28-year-old woman, gravida 0 para 0. She had regular menses.

**Figure 1:**
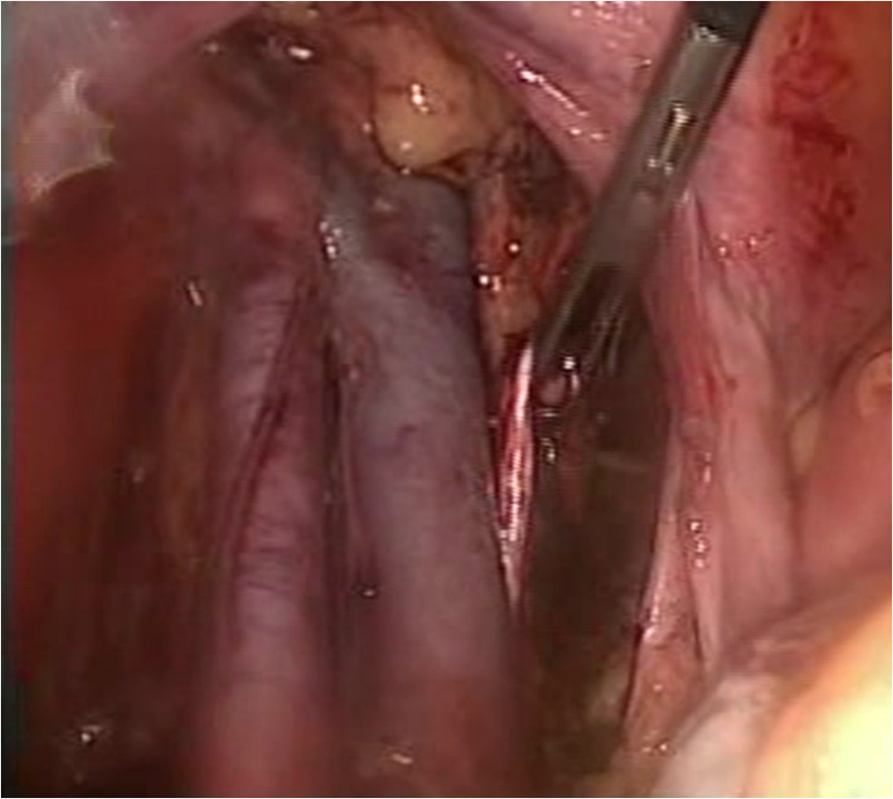
Left obturator nerve being transected during lymphadenectomy.

**Figure 2:**
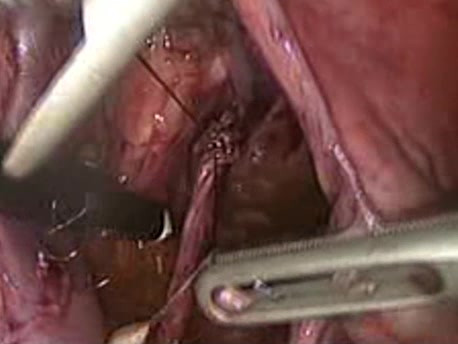
Intraoperative epineural end-to-end anastomosis.

**Figure 3:**
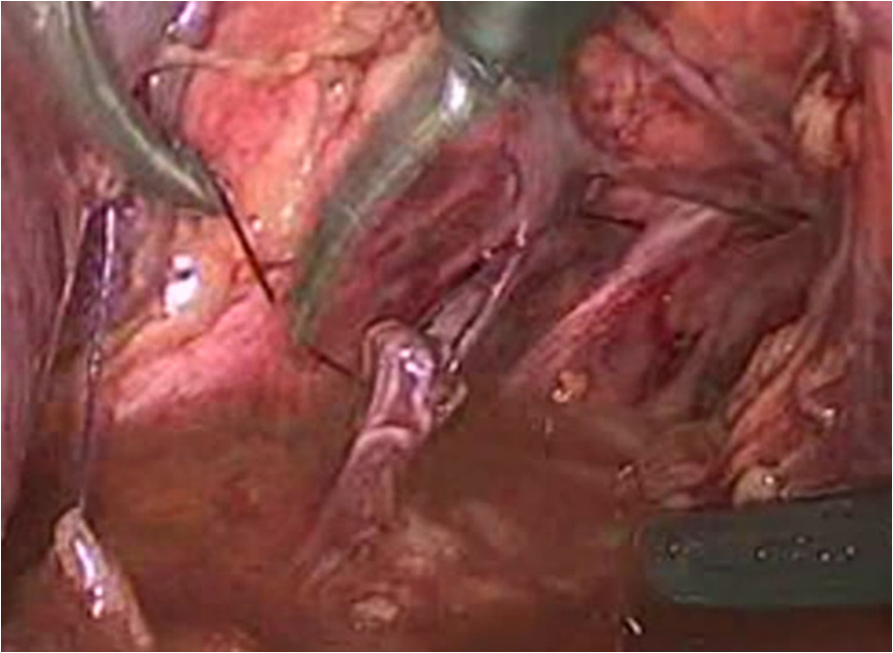
View of the sutured nerve in the left obturator fossa.

Her first preventive gynecologic checkup, including cytologic smear and gynecologic examination dated back two years, and was negative for pre-cancerous or cancerous lesions. A Pap smear was obtained three months before surgery. It showed positivity for a high-grade squamous intraepithelial lesion. One month later, a biopsy was performed: “undifferentiated squamous carcinoma of the cervix”. She was eventually referred to our centre where a colposcopy was scheduled: “vulva and vagina negative. SCJ visualized. Thick acetowhite epithelium surrounding the orifice. Irregular punctation h 7 (<0.5 cm). Iodonegative. Endocervical curettage: presence of atypical cells with a morphology resembling a carcinoma”. Positivity for HPV 18 was also present. Rectovaginal examination with the patient under anesthesia revealed an exophytic lesion extending from the inner cervix with a diameter of 2.5 cm. Magnetic resonance imaging (MRI) of the abdomen confirmed the presence of an expansive exocervical formation located posteriorly with a maximum diameter of 2 cm. The lesion did not appear to involve the vagina or the contiguous anatomic structures. Iliac lymph nodes were bilaterally subcentrimetic. MRI staging showed FIGO stage IB. Cystoscopy demonstrated no infiltration of the bladder. A biopsy specimen revealed a moderately differentiated squamous cell carcinoma. Metastatic workup, including computed tomography of the thorax and abdomen, was negative for distant metastasis. The patient expressed a desire of preserving fertility. She was informed of optional fertility-sparing surgery to which she gave consensus. The procedure included bilateral radical pelvic lymphadenectomy and cervical conization. A four-port transperitoneal laparoscopic approach was used in order to remove pelvic lymph nodes. After completion of the procedure on the left side, anatomical landmarks were checked and it became evident that the obturator nerve was sharply transected over a distance of 5 mm. Laparoscopic lymphadenectomy was completed uneventfully. The Department of Neurosurgery was consulted intraoperatively. Careful inspection revealed that the nerve was transected cleanly without any fraying of the edges. Because the resected portion of the nerve was only 5 mm, tension-free reattachment of the edges of the nerve seemed possible without further mobilization. The obturator nerve edges were oriented and laparoscopically re-approximated end-to-end with five 6/0 braided polyester epineural sutures to achieve a tension-free anastomosis (Figures 1, 2 and 3). The gynecologic surgeon completed the nerve repair. Total operative time was 5½ hours, and blood loss was 150 mL. Early postoperative course was uneventful.

Histologic examination revealed poorly differentiated squamous cell carcinoma of the cervix. Lymph-vascular space invasion was negative. Tumor stage was pT1B, G3, pN0 (pelvic nodes 0/35), IB (ajcc 2010). Postoperatively, the patient did not exhibit any clinically apparent loss of adductor function or any other neurologic deficiency at the neurosurgeon examination. Therefore, no further neurologic examination, electromyography or specific physical therapy was advised at that time. Neurologic examination at the three-month follow-up revealed no motor deficit of adduction of the leg, and no evidence of a sensory deficit of the obturator nerve area. Electromyography of the adductor magnus muscle on the right demonstrated no pathologic spontaneous activity, but extensive polyphasic muscle action potentials, suggesting reinnervation.

The obturator nerve originates from the anterior division of the ventral rami of the second, third and fourth lumbar spinal nerves within the psoas major muscle, resulting from the unification of the rami. It descends through the psoas muscle to emerge from its medial border at the pelvic brim. It runs over the pelvic brim into the lesser pelvis, curving anteroinferiorly and following the lateral pelvic wall to pass through the obturator foramen in which it divides into anterior and posterior branches. The anterior branch innervates the adductor longus, gracilis and adductor brevis muscles and also gives off sensory fibers that innervate the skin and fascia of the medial aspect of the midthigh. The posterior division pierces and innervates the obturator externus. Then it runs between the adductor brevis and magnus muscles and splits into a motor branch that supplies adductor magnus and a sensory branch to the knee joint to supply the articular capsule, cruciate ligaments and synovial membrane of the knee joint. The posterior branch occasionally innervates the adductor brevis [1,2,4,5]. Obturator nerve injury is rare, and is most frequently associated with a gynecologic or urologic procedure for cancer, endometriosis or prolonged lithotomy positioning [6]. Neurotmesis of the obturator nerve has been rarely reported as a surgical complication in gynecologic surgery [7].

## Conclusions

The obturator nerve is an important landmark during pelvic lymph node dissection. During pelvic lymphadenectomy, the obturator nerve can undergo indirect thermal injury or direct complete division as a result of blunt or sharp dissection. When the lesion appears uncomplicated, as in our case, immediate laparoscopic repair is feasible and safe.

## Consent

Written informed consent was obtained from the patient for publication of this Case report and any accompanying images. A copy of the written consent is available for review by the Series Editor of this journal.

## Competing interests

The authors declare that they have no competing interests.

## Authors’ contributions

ER conceived of the study, collected data and drafted the manuscript. MJ participated in the design of the study. PM participated in imaging editing and collection. MS helped in drafting the manuscript. AF corrected and revised the manuscript. BK performed the surgery and participated in study design. MM participated in coordination and helped to draft and edit the manuscript. All authors read and approved the final manuscript.
